# Insights from the molecular docking analysis of SGLT2 and FIMH to combat uropathogenicity

**DOI:** 10.6026/973206300181044

**Published:** 2022-11-30

**Authors:** Wesam H Abdulaal, Muhammed A Bakhrebah, Majed S Nassar, Ibrahim Abdullah Almazni, Wael Abdullah Almutairi, Zuhair S Natto, Amin K Khattab

**Affiliations:** 1 Department of Biochemistry, Faculty of Science, Cancer and Mutagenesis Unit, King Fahd Medical Research Center, King Abdulaziz University, Jeddah 21589, Saudi Arabia; 2 Centre for Artificial Intelligence in Precision Medicines, King Abdulaziz University, Jeddah 21589, Saudi Arabia; 3 Life Science and Environment Research Institute, King Abdulaziz City for Science and Technology (KACST) Riyadh 1144, Saudi Arabia; 4 Department of Clinical Laboratory Sciences, Faculty of Applied Medical Sciences, Najran University, P.O. Box 1988, Najran 61441, Saudi Arabia; 5 Department of Respiratory Services, Ministry of National Guard Hospital and Health Affairs (MNGHA) P.O. box 22490, kingdom of Saudi Arabia; 6 Department of Dental Public Health, Faculty of Dentistry, King Abdulaziz University, Jeddah 21589, Saudi Arabia; 7 Quality and Outcome Control Management, General Directorate of Health Affairs-Madina Region, Ministry of Health, Madina 32000, Saudi Arabia

**Keywords:** SGLT2 inhibitors, urinary tract infections, FimH, natural compounds

## Abstract

SGLT2 inhibitors are a novel class of FDA approved anti-diabetes drugs. They act by blocking the SGLT2 protein, which prevents glucose reabsorption, leading in enhance glucose excretion and lower blood glucose levels. In diabetic patients, SGLT2 inhibitors have been linked to urinary tract infections (UTIs). Therefore, the development of novel SGLT2 inhibitors with no adverse effects is a need of time. With this purpose, in this study, 48164natural compounds from ZINC database were screened targeting both the SGLT2 and FimH protein using insilico approaches. FimH has been discovered as a promising target for preventing and treating UTIs. The hit compounds ZINC69481892, ZINC1612996, and ZINC4039265 exhibited strong binding with both SGLT2 and FimH with binding energies values of -9.88, -8.96, and -10.57 kcal/mol for SGLT2, and -7.86, -7.01, and -8.92 kcal/mol for FimH, which is higher than that of controls (-6.78 kcal/mol (Empaglifozolin for SGLT2) and -5.14 kcal/mol (Heptyl α-d-mannopyranoside for FimH)). Hits were found to bind with key residues of both SGLT2 and FimH protein. In addition, physiochemical properties showed that these compounds have good drug-likeness properties. Therefore, we anticipate that if these compounds are investigated further, might be potential SGLT2 inhibitors with less uropathogenic adverse effects.

## Background:

Diabetes is among the world's quickest growing ailments, with 693 million individuals expected to be affected by 2045. Type 2 Diabetes Mellitus (T2DM), the most prevalent metabolic disease, is characterized majorly by a combination of two major factors: impaired insulin production by pancreatic -cells and a failure of insulin-sensitive tissues to respond adequately to insulin. In T2DM, primary care includes pharmacological treatment using insulin sensitizers, insulin secretagogues, alpha-glucosidase inhibitors, and the more recently developed incretin-based treatments and sodium-glucose cotransporter-2 (SGLT2) inhibitors. SGLT2 inhibitors are a novel class of medications that are used to treat T2DM. They work by inhibiting the SGLT2 protein, which is involved in preventing glucose reabsorption, resulting in increased glucose excretion and reduced the blood glucose levels [1]. These inhibitors increase the incidence of urinary tract infections (UTIs) by increasing urine glucose levels [2]. In diabetic patients, pharmacologically induced urine glucose levels using SGLT2 inhibitors may support bacterial growth and increase the risk of UTIs [3] [4]. The FDA warned in December 2015 that SGLT2 inhibitors might cause severe UTIs [5,6]. Empagliflozin and canagliflozin are the recommended medicines for T2DM patients with existing cardiovascular disease [7,8]. From March 2013 to October 2014, a public health advisory based in the United States reported 19 occurrences of deadly renal or blood infection. These cases were linked to a UTI caused by SGLT2 inhibitor use [5]. Bacterial attachment to urothelial cells is a vital step in the establishment of UTI, which is mediated by 0.1-2 µm long proteinaceous filaments on the bacterial surface known as type 1 pili [9]. Type 1 pili are made up of multiple subunits, the most important of which is FimH (adhesion protein), which plays a key role in pathogenesis of Escherichia coli in the urinary tract. E. coli cell invasion and adhesion to mannose-containing glycoproteins are both mediated by this protein [10]. The ability to colonize and invade the bladder epithelium, as well as the ability to form intracellular bacterial communities, has been proven to be important factors in E. coli pathogenicity. Here, we used an in-silico technique to screen natural compounds from the ZINC database targeting both the SGLT2 and FimH protein that might have less uropathogenic adverse effects.

## Methodology:

### Protein preparation:

3-D structure of FimH (PDB ID: 4AV5) was obtained from the protein data bank, however,3-D structure of SGLT2 was predicted using the SWISS-MODEL Workspace after collecting its amino acid sequence from Uniprot (P31639).

### Database collection:

ZINC is a free available chemical database (http://zinc20.docking.org/) that is being used to find possible leads [11]. In this study, 48164 natural compounds from ZINC database were retrieved in SDF format, then minimized and prepared for screening using the "ligand preparation" tool in DS 2020.

### Virtual screening and molecular docking:

Using AutoDock vina (version 1.1.2), the prepared library was screened against SGLT2 and FimH protein to find the potential leads. Autodock 4.2 was used to perform automated molecular docking of hits with SGLT2 and FimH protein [12]. The Autogrid program was employed to generate a grid map of 40 x 40 x 40 with 0.375 spacing to calculate the binding energies (BEs) between the ligand-protein complexes. The most promising binding pose was determined to be the one with the high negative BE value.

### Pharmacokinetics and physiochemical properties prediction:

Pharmacokinetics, toxicity and physiochemical properties was predicted for the best three screened compounds by employing Datawarrior tools [13] and ProTox-II webserver [14].

## Result and Discussion:

SGLT2 inhibitors are a novel class of anti-diabetes drugs [15]. In diabetic patients, these inhibitors have been linked to UTIs and genital infections [16, 17]. Therefore, the development of novel SGLT2 inhibitors with no adverse effects is a need of time. In this study, 48164 natural compounds were screened targeting the catalytic sites of both the SGLT2 and FimH protein. FimH has been discovered as a promising target for preventing and treating UTIs [18]. The AutoDock vina (version 1.1.2) has been used to screen the prepared library of compounds, and the top 15 compounds identified by the VS against both target proteins are listed in Table 1(see PDF) Among the screened compounds, the hits ZINC69481892, ZINC1612996, and ZINC4039265 were dock to the catalytic site of SGLT2 and FimH and showed strong binding with both proteins. ZINC69481892 bind with the Asn75, His80, Phe98, Glu99, Ala102, Lys154, Val286, Ser287, Trp289, Tyr290, Trp291, Phe453, Ile456, Gln457, and Ser460 residues of SGLT2 (Figure 1a - see PDF); while Ser74, Asn75, Ile76, Gly77, Ser78, Gly79, His80, Leu84, Glu99, Lys154, Asp201, Gln204, Thr205, Tyr290, Trp291, Met328, Ala389, Ser392, Ser393, Ser396, Ile397, Phe453, Ile456, and Gln457 residues of SGLT2 were interacted with ZINC1612996 (Figure 1b - see PDF). Further, ZINC4039265 bind with Ala73, Ser74, Asn75, Ile76, Gly77, Ser78, His80, Phe98, Lys154, Asp158, Gln204, Thr205, Ile208, Val286, Tyr290, Trp291, Ser393, Ala389, Phe453, Ile456, and Gln457 residues of SGLT2 (Figure 1c - see PDF). BE of ZINC69481892, ZINC1612996, and ZINC4039265 with SGLT2 interactions were noted be -9.88, -8.96, and −10.57 kcal/mol, respectively (Table 2 - see PDF).

The residue at position 457 has a significant impact on SGLT1 protein function. Residue 457 (i.e., Gln457) in SGLT1 has been demonstrated to interact directly with sugar during its reabsorption [19]. Both SGLT1 and SGLT2 contain glutamine at position 457, and mutation in this spot (Gln457) induces glucose-galactose malabsorption [20]. In line with this, hit compounds were found to interact with Gln457 residue in this study, which is thought to impede SGLT2 function. Empagliflozin was authorized by the FDA for use in T2DM treatment in 2014 [21], and has used as a control against SGLT2 in this study. Asn75, His80, Lys154, Tyr290, Trp291, Phe453, and Ile456 were the common binding residues of SGLT2 with the hit compounds and empagliflozin (Figure 1a-1d - see PDF). FimH adhesin promotes E. coli colonization and biofilm development on the human bladder's cell surface [22]. Hence, FimH has been identified as a potential curative target for the management of UTIs [23]. Interestingly, ZINC69481892 interacted with Phe1, Ile13, Asp47, Tyr48, Thr51, Ile52, Asp54, Gln133, Asn135, Tyr137, Asp140, and Phe142 residues of FimH (Figure 2a - see PDF); while Ile13, Tyr137, Asn138, Ser139, Asp140, Asp141, and Phe142residues of FimH were found to interacted with ZINC1612996 (Figure 2b - see PDF). In addition, ZINC4039265 interacted with Phe1, Ile13, Gly14, Asp47, Tyr48, Ile52, Gln133, Tyr137, Asn138, Asp140, and Phe142residues of FimH (Figure 2c - see PDF). Tyr48 is important in the regulation of FimH-antagonist binding. Interestingly, ZINC69481892 and ZINC4039265 have found to bind with Tyr48 residue of FimH. BE of ZINC69481892, ZINC1612996, and ZINC4039265 with FimH were found to be -7.86, -7.01, and -8.92 kcal/mol, respectively (Table 2 - see PDF). Heptyl α-d-mannopyranoside is a FimH antagonist [24], and has been used as the reference compound against FimH in this study. Ile13, Asp140, and Phe142 were the common interaction residues of FimH with the hit compounds and Heptyl α-d-mannopyranoside (Figure 2a-2d - see PDF). Phe1, Asn46, Asp47, Asp54, Glu133, Asn135, Asp140, and Phe142 are the key FimH binding pocket residues, and mutations in these individual key residues influence FimH function and reduce its pathogenicity [25, 26]. Accordingly, present study showed that hit compounds bind with these FimH residues.

In 2002, 3D structure of FimH complex with α-D-mannose bound to the carbohydrate-binding domain (CRD) was published. This structure showed which amino acid residues were relevant for mannose binding in CRD. Mannose binds with residues in the negatively charged pocket of CRD domain by the ten hydrogen bonds. All -OH groups of the mannose sugar bind with Phe1, Asn46, Asp47, Asp54, Gln133, Asn135, Asp140 and Phe142 amino acids. In this complex, the hydrophobic residues namely Ile13, Tyr48, Ile52, Tyr137 and Phe142 surrounded the mannose-binding pocket. Tyr48 and Tyr137 side chains are situated in order to form a "tyrosine gate" [25]. Interestingly, in this study, FimH residues Phe1, Ile13, Asn46, Asp47, Tyr48, Ile52, Asp54, Gln133, Asn135, Asp140 and Phe142 contributed in interaction with the hit compounds (ZINC69481892, ZINC1612996, and ZINC4039265). Insufficient pharmacokinetics and accessibility, as well as effectiveness and toxicity, have been responsible for a number of drug development failures. Gastrointestinal absorption and brain permeability are two pharmacokinetic parameters that should be considered at various stages of the pharmaceutical development process. The top three compounds (ZINC69481892, ZINC4039265, and ZINC1612996) contain almost all of the attributes needed to be future drug-like molecules, as per their physicochemical properties, and toxicity evaluation (Table 3 and 4 - see PDF). Toxic doses are usually expressed as LD50 values in mg/kg body weight. The median lethal dose (LD50) is the amount of a substance that causes 50% of test subjects to die. The ProTox-II webserver estimated the LD50 values for ZINC69481892, ZINC4039265 and ZINC1612996 to be 2000, 3000, and 765 mg/kg, respectively.

## Conclusion:

This study used in silico art of technique to identify natural SGLT2 and FimH inhibitors from the ZINC database. The hit compounds (ZINC69481892, ZINC1612996, and ZINC4039265) had high binding affinity with both SGLT2 and FimH relative to the reference compounds. Conversely, the known SGLT2 inhibitor had less interaction with FimH. Therefore, we anticipate that if these hits are investigated further, might be potential SGLT2 inhibitors with less uropathogenic adverse effects.
